# Occult Hepatitis B Infection in End-Stage Renal Disease Patients Starting Maintenance Hemodialysis at a Tertiary Care Hospital: A Descriptive Cross-sectional Study

**DOI:** 10.31729/jnma.5723

**Published:** 2021-04-30

**Authors:** Shailendra Shrestha, Pratap Sagar Tiwari, Bikram Pradhan

**Affiliations:** 1 Department of Internal Medicine, Nobel Medical College Teaching Hospital, Biratnagar, Nepal; 2 Department of Gastroenterology and Hepatology, BP Koirala Institute of Health Sciences, Dharan, Nepal

**Keywords:** *end-stage renal disease*, *hemodialysis*, *hepatitis B*

## Abstract

**Introduction:**

Occult hepatitis B infection is defined as the presence of the hepatitis B virus deoxyribonucleic acid in liver tissues and/or serum in the absence of serum hepatitis B Virus surface antigen. The prevalence of occult hepatitis B infection in end-stage renal disease patients is largely unknown. The aim of the study is to determine the prevalence of occult hepatitis B infection in the hemodialysis population starting maintenance hemodialysis.

**Methods:**

A descriptive cross-sectional study was conducted in the department of Internal Medicine of a tertiary care hospital. Convenience sampling method was used; 50 consecutive end-stage renal disease patients, who started maintenance hemodialysis from March 2019 to March 2020, were enrolled in the study. The study was approved by the Institutional Review Committee of the hospital (reference number: 351/2019). Statistical Package for Social Sciences version 26.0 was used for statistical analysis.

**Results:**

The mean age of the patients was 50.34±12.65 years, and 42 (84%) were male. About 4 (8%) patients were diagnosed having occult hepatitis B infection, 3 (6%) of them were seropositive and 1 (2%) seronegative. About 41 (82%) patients had no history of hepatitis B vaccination series before starting hemodialysis; 36 (72%) had anti-hepatitis B surface antibody titre <10 mIU/ml. About 44 (88%) patients received a blood transfusion during their hemodialysis sessions and 14 (28%) patients had a history of receiving hemodialysis at other centres.

**Conclusions:**

Our study demonstrated a high prevalence of occult hepatitis B infection among end-stage renal disease patients starting hemodialysis.

## INTRODUCTION

Occult hepatitis B infection (OBI) is defined as the presence of the hepatitis B virus (HBV) deoxyribonucleic acid (DNA) in liver tissues and/or serum in the absence of serum HBV surface antigen (HBsAg).^1,2^ OBI can be classified into two groups: seropositive OBI, where anti-hepatitis B core antibody (anti-HBc) is positive with or without a positive anti-hepatitis B surface antibody (anti-HBs), and seronegative OBI, where both anti-HBc and anti-HBs are negative.^3-5^

The prevalence of OBI in hemodialysis (HD) patients varies from 0 to 58% depending upon the prevalence of HBV infection in the general population.^6,7^

The present study was carried out to determine the prevalence of OBI in end-stage renal disease (ESRD) patients starting maintenance hemodialysis (MHD) at tertiary care teaching hospital of Eastern Nepal.

## METHODS

A descriptive cross-sectional study in the department of Internal Medicine of Nobel Medical College Teaching Hospital (NMCTH) among consecutive ESRD patients starting MHD from March 2019 to March 2020 was enrolled in the study. The study was approved by the Institutional Review Committee of the hospital (NMCTH IRC reference number: 351/2019). The convenience sampling method was used. All consecutive ESRD patients starting MHD from March 2019 to March 2020 giving written informed consent were enrolled in the study. While ESRD patient not giving written consent were excluded from the study. The sample size was calculated using the formula,

$$\begin{array}{ll} \text{n} & ={\text{Z}}^{\text{2}}\times \text{p}\times \text{q}/{\text{e}}^{\text{2}}\\ & ={\left(1.96\right)}^{2}\times (0.5)\times (0.5)/{\left(0.14\right)}^{\text{2}}\\ & =49 \end{array}$$

where,

n = sample size,
Z = 1.96 at 95% Confidence Interval
p = prevalence, 50%
q = 1-p
e = margin of error, 14%

The calculated sample size is 49. About 50 patient were included in the study.

Written informed consent was taken from all participants prior to inclusion in the study. Patients who were HBsAg, anti-hepatitis C antibody (anti-HCV), and human immunodeficiency virus (HIV) negative on initial screening tests were included in the study. HBsAg, anti-HCV, and HIV serology positive patients were excluded from the study. Serological tests for HBsAg, anti-HCV and HIV are routinely repeated at an interval of every three months in our centre as a protocol.

The demographic and clinical characteristics of the study population were digitally recorded at the time of enrollment in our MHD program. Serum samples of the study patients were analyzed at National Reference Lab, Dr. Lal Path Labs, New Delhi, India for

(1) HBV DNA, quantitative test [real time polymerase chain reaction (PCR), Taqman technology],

(2) total anti-HBc level,

(3) anti-HBs level and

(4) HCV ribonucleic acid (RNA), quantitative [real time PCR, Taqman technology].

The linear reporting range of HBV DNA assay is 20–1.7 × 100000000IU/ml (conversion factor: 1IU/ml = 5.82 copies/ml) and that of the HCV RNA assay is 21–1.77 × 10000000IU/ml in this laboratory. Anti-HBc, a total of >1 was taken as reactive and indicative of prior infection. Anti-HBs titre of >10mIU/m was taken as protective and indicative of either prior hepatitis B vaccination or prior infection. Samples for the above tests were sent within one month of initiating dialysis at our centre.

Occult hepatitis B infection (OBI) was defined in our study as having detectable HBV DNA in the serum in the absence of HBsAg, with or without reactive anti-HBc and anti-HBs antibody.^2^ We classified patients as having “prior hepatitis B infection” if HBV DNA in serum was not detectable but anti-HBc and/or anti-HBs were reactive. Patients with only positive anti-HBc but negative HBsAg and negative anti-HBs were labelled as “isolated anti-HBc positive".^2-4^

Statistical Package for Social Sciences (SPSS) version 26.0 was used for statistical analysis. Data were reported as mean±standard deviation (SD) and range, or number (n) and percentage (%), whatever applicable.

## RESULTS

A total of 50 consecutive new ESRD patients who were started on maintenance hemodialysis during the study period were included in the study. About 4 (8%) patients were diagnosed having occult hepatitis B infection, 3 (6%) of them were seropositive and 1 (2%) seronegative. The mean age of the patients was 50.34±12.65 years (range 22-78), and 42 (84%) of them were male. The most common cause of ESRD was diabetes mellitus 26 (52%), followed by chronic glomerulonephritis 16 (32%) (Table 1).

**Table 1:** Baseline characteristics of the patients (n = 50).

Variables	Mean±SD (range)	n (%)
Age (years)	50.34±12.65 (22-78)	
Sex		
Male		42 (84)
Female		8 (16)
Duration of CKD^*^ (months)	19.56±24.28 (1-84)	
Cause of CKD		
DM†		26 (52)
CGN‡		16 (32)
HTN§		6 (12)
ADPKD||		2 (4)
Initiation of MHD was emergency or elective		
Emergency		41 (82)
Elective		9 (18)
Indication of initiating MHD		
Pulmonary edema		36 (72)
Early uremic symptoms		9 (18)
Uremic encephalopathy		3 (6)
Metabolic acidosis		1 (2)
Uremic pericarditis		1 (2)
Was AVF ^¶^ made prior to the initiation of maintenance haemodialysis		
No		40 (80)
Yes		10 (20)
Vascular access at the time of first HD		
Temporary HD catheters		44 (88)
RC ** AVF		5 (10)
BC †† AVF		1 (2)
Haemoglobin (gram/dl) at the time of initiation of maintenance haemodialysis	6.89±1.86 (3-11)	

* CKD: chronic kidney disease

† DM: diabetes mellitus

‡ CGN: chronic glomerulonephritis

§ HTN: hypertension

|| ADPKD: adult polycystic kidney disease

¶ AVF: arteriovenous fistula

** RC: radiocephalic

†† BC: brachiocephalic.

We identified four patients (8%) as having OBI. We labelled three (6%) patients as seropositive OBI and one (2%) as seronegative OBI (Table 2).

**Table 2:** Characteristics of four patients with OBI.^*^

S. N.	Age	Sex	BT ^†^ during HD	HD at other centre	Hepatitis B vaccination status	HBVDNA (IU/ml)	Anti-HBc (total)	Anti-HBs (mIU/ml)	HCVRNA (IU/ml)	Impression
1	27	female	yes	yes	Yes, series completed	27	2.89	289	not detected	seropositive OBI
2	70	female	yes	no	no	41	0.10	0.17	not detected	seronegative OBI
3	46	male	yes	no	no	48	7.27	5.38	not detected	seropositive OBI
4	39	male	yes	yes	no	115	1.57	0.06	not detected	seropositive OBI

* OBI: occult hepatitis B infection

† BT: blood transfusion.

We also identified six patients (12%) who had serological evidence of “prior hepatitis B infection” without a detectable HBV DNA in their serum. Five of them had both anti-HBc and anti-HBs titer reactive (past infection with natural immunity) and one patient had only anti-HBc titer reactive (isolated anti-HBc positive). Hence by definition, we had three patients as ‘isolated anti-HBc positive’, two of them were seropositive OBI and one suggestive of prior HBV infection but without OBI and without natural immunity.

None of the patients in our cohort had HCV RNA detected in serum.

None of the patients in our study, on three monthly intervals of serological tests, had seroconversion to HBsAg positive and/or anti-HCV positive during the study period.

While mentioning the potential risk factors for OBI, six patients (12%) and three patients (6%) had a history of fully completed and partially completed hepatitis B vaccination history respectively, however, all of them had an anti-HBs titre of >10 mIU/ml. Forty-one patients (82%) had not started the hepatitis B vaccination series before starting hemodialysis; 36 (72%) of them had anti-HBs titre <10 mIU/ml. Five out of 41 patients ( with no prior history of hepatitis B vaccination) had anti-HBs titre >10 mIU/ml because of the prior HBV infection (evidenced by reactive anti-HBc, as described later). Forty-four (88%) of patients received a blood transfusion during their hemodialysis sessions and 14 (28%) of patients had a history of receiving HD at other centres too (Table 3).

**Table 3:** Potential Risk factors for OBI * (n = 50).

Risk factors	n (%)
Hepatitis B vaccination status	
Vaccination course completed	6 (12)
Vaccination course started but not completed	3 (6)
Vaccination course not started yet	41 (82)
Anti HBs titre	
<10 mIU/ml	36 (72)
>10 mIU/ml	14 (28)
Blood transfusion during hemodialysis	44 (88)
History of hemodialysis at other centre	14 (28)

## DISCUSSION

To the best of our knowledge, no study has been done in Nepal about the prevalence of OBI either in the general population or in high-risk groups. Ours is the first study in Nepal looking for the prevalence of OBI in hemodialysis patients. We found that the prevalence of OBI in our hemodialysis patients was 8%. This is higher than the prevalence of chronic carriers of HBV in the general population in Nepal.

The prevalence of HBV and HCV infection, including OBI, in the hemodialysis population, depends upon its prevalence in the general population.^4,5,7^ Depending upon the prevalence of HBV carriers in the general population, Nepal has been considered a low endemic area (<2 %), which is in sharp contrast to its two neighbouring countries China, and India.^8^ China is considered a high endemic area (≥8%) with a prevalence of about 10% and India with a prevalence of about 5% is considered an intermediate endemic area (2-7%).^8^ The prevalence of chronic carriers of HBV in the general population of Nepal has been found to be about 1% in different studies conducted at different time periods.^9,10^ The low prevalence of HBV in the general population of Nepal has been attributed to various factors like a low rate of perinatal transmission and its predominant spread by horizontal transmission among the adolescent age group.^8-10^ In 2019 World Health Organization (WHO) announced that Nepal, along with Bhutan, Bangladesh and Thailand have become the first countries in WHO South-East Asia Region to achieve Hepatitis B control, with the prevalence of the disease dropping to less than one percent among five-year-old children.^11^

We found only three studies in Nepal addressing HBV and HCV in the hemodialysis population.^12-14^ The first study, published in 1998, showed that one out of 22 HD patients (5%) had HCV RNA detected and the patient had received more blood transfusions than the patients who did not have HCV RNA.^12^ In the second study conducted at the National Academy of Medical Sciences, Bir Hospital, and ShreeBirendra hospital, Kathmandu in 2009, 54 ESRD patients on MHD and who were seronegative for HBV, HCV, and HIV were included; seroconversion of HBV, HCV, and HIV was nil during 18 months of follow up.^13^

The third study was conducted at Patan Academy of Health Sciences, Lalitpur in 2015;12 (6.9%) out of 173 patients seroconverted to HCV during the study period of 5 years.^14^

The concentration of HBV DNA in OBI is low, usually less than 200 IU/ml (about 1,000 copies/ml).^1,4,5^ In our study also all four patients have an HBV DNA level of less than 200IU/ml. Three out of four OBI patients in our study were seropositive OBI; indeed seropositive OBI contributes to about 80% of total OBI cases.^1,3-5^

The risk factors for acquiring HBV and HCV infections, including OBI, in HD patients are breech in the standard infection prevention measures, not taking hepatitis B vaccination prior to starting MHD, anti-HBs level < 10IU/ml, multiple blood transfusions, and doing HD at multiple centres.^2-4^ In our study 41 patients (82%) had no history of taking hepatitis B vaccination and 36 patients (72%) had an anti-HBs titer of <10IU/ml. Widespread implementation of hepatitis B vaccination in CKD patients has shown to decrease the incidence and prevalence of HBV infection in HD patients. Furthermore, hepatitis B vaccination early in the course of CKD generates a better immune response and a high likelihood of achieving anti-HBs titer of >10IU/ml.^15^ Mean Hemoglobin at the time of initiation of MHD in our study was 6.89±1.86 (3-11)gram/L and 44 patients (88%) had received a blood transfusion during HD sessions. This could have been avoided by correcting anaemia in the pre-ESRD phase of the patients by using iron and/or erythropoiesis-stimulating agents.^16^ Early identification of CKD, referral to nephrologists when patients reach CKD stage 3, and a good pre ERSD care of the patients decreases not only the risk of acquiring blood-borne infections but also improves overall morbidity and mortality.^17^

One of our seropositive OBI patient had an anti-HBs titer of 289 mIU/ml. The possible explanation for the protective titer of anti-HBs in this particular patient is that some individuals who recover from HBV infection and produce neutralizing anti-HBs may continue to replicate HBV DNA at low levels that are detectable for years in the liver and/or peripheral blood. In such cases, the virus is either complexed with anti-HBs, or free in the circulation, or both forms might be present.^18^ Another possible reason may be that she also had a history of a full course of hepatitis B vaccination in past. We speculate that she first acquired OBI (and hence her HBV DNA level was 27 IU/ml and anti-HBc, the total was 2.89) but it was undetected. Later during the course of her CKD, she received a full course of hepatitis B vaccination and hence developed a protective titer of anti-HBs.

We diagnosed three out of 50 patients (6%) as having “isolated anti-HBc positive” i.e. presence of anti-HBc in the absence of HBsAg and anti-HBs.^1,2,4^ Isolated anti-HBc may sometimes represent OBI as shown in our study, where two out of three such patients were having detectable HBV-DNA level. It is, in general, suggested that all “isolated anti-HBc positive patients” should undergo PCR tests to detect HBV DNA.^3-5^

None of our patients had seroconversion to positive HBsAg and/or positive anti-HCV during the study period; however, it might be because of the short duration of our study. The seroconversion rate of HBV and HCV infection in HD patients has been found to be associated with the longer duration on HD, level of training of HD staff, presence of protocols for infected patients, and other comorbid conditions.

The limitations of our study are that it was a single centre study, was restricted to one geographical region, and included a small number of patients over a small period. Hence the results may not represent all the dialysis centres of Nepal. However, we strongly suggest that nation wise similar study needs to be in Nepal including a large number of ESRD patients from different hemodialysis centres.

## CONCLUSIONS

Our study demonstrated a high prevalence of occult hepatitis B infection among maintenance hemodialysis patients at a tertiary level teaching hospital in Eastern Nepal. Our study should pave the way to conduct similar studies in multiple hemodialysis units of Nepal involving a large number of patients.
